# MiR-107 Regulates Adipocyte Differentiation and Adipogenesis by Targeting Apolipoprotein C-2 (*APOC2*) in Bovine

**DOI:** 10.3390/genes13081467

**Published:** 2022-08-17

**Authors:** Xuefeng Wei, Xue Zhao, Xinyue Shan, Yunchang Zhu, Shuzhe Wang, Hong Chen, Hui Li, Yun Ma

**Affiliations:** 1 College of Life Sciences, Xinyang Normal University, Xinyang 464000, China; 2 School of Agriculture, Ningxia University, Yinchuan 750021, China; 3 Shaanxi Key Laboratory of Molecular Biology for Agriculture, College of Animal Science and Technology, Northwest A&F University, Yangling 712100, China; 4 State Key Laboratory for Conservation and Utilization of Subtropical Agro-Bioresources, College of Animal Science and Technology, Guangxi University, Nanning 530004, China

**Keywords:** bovine, miR-107, *APOC2*, adipocyte differentiation, adipogenesis

## Abstract

Adipogenesis is a complex and precisely orchestrated process mediated by a series of adipogenic regulatory factors. Recent studies have highlighted the importance of microRNAs (miRNAs) in diverse biological processes, most specifically in regulating cell differentiation and proliferation. However, the mechanisms of miRNAs in adipogenesis are largely unknown. In this study, we found that miR-107 expression was higher in bovine adipose tissue than that in other tissues, and there was a downregulation trend during adipocyte differentiation. To explore the function of miR-107 in adipocyte differentiation, agomiR-107 and antiagomiR-107 were transfected into bovine adipocytes, respectively. Oil Red O staining, CCK-8, EdU assays, RT-qPCR, and Western blotting were performed, and the results showed that overexpressed miR-107 significantly suppressed fat deposition and adipocyte differentiation, while knockdown of miR-107 promoted fat deposition and adipocytes differentiation. In addition, through bioinformatics analysis, luciferase reporter assays, RT-qPCR, and Western blotting, we identified apolipoprotein 2 (*APOC2*) as a target of miR-107. Transfection of siRNA-*APOC2* into adipocytes led to suppression in adipocyte differentiation and proliferation, suggesting a positive role of APOC2 in bovine lipogenesis. In summary, our findings suggested that miR-107 regulates bovine adipocyte differentiation and lipogenesis by directly targeting *APOC2*, and these results. These theoretical and experimental basis for future clarification of the regulation mechanism of adipocyte differentiation and lipogenesis. Moreover, for the highly conserved among different species, miR-107 may be a potential molecular target to be used for the treatment of lipid-related diseases in the future.

## 1. Introduction

Excessive accumulation of fat is an important reason for obesity in humans, which causes a series of obesity-related diseases. However, adipose tissue has important functions for mammals to maintain body temperature and provide energy. Obesity has focused attention on adipose tissue and the development of adipocytes, which is known as adipogenesis. Adipogenesis is a precisely orchestrated and complex process mediated by a network of adipogenic regulatory factors. CCAAT/enhancer binding proteins (C/EBPα) and Peroxisome proliferator activated receptor gamma (PPARγ) have emerged as crucial regulators of adipogenesis [1]. C/EBPα and PPARγ coordination regulates the adipogenic differentiation progress. Recent studies have indicated that when PPARγ is absent, other factors, which may be positive or negative regulators of adipocyte development, cannot drive adipogenesis alone [2,3].

microRNAs (miRNAs; 17–23 nucleotides), one of the non-coding small RNAs, play an important role in the regulation of cell proliferation, differentiation, and apoptosis by targeting mRNAs to inhibit their expression. miR-27a [4] and miR-181 [5] regulate adipocyte differentiation, while miR-1, miR-206 [6], miR-133a [7], miR-378a-3p [8], miR-103, and miR-144 [9] regulate myoblasts proliferation and differentiation. However, the molecular mechanisms of most miRNAs are mainly unknown. miR-107 has been reported to play an important role in the differentiation of myocytes [10] and endothelial progenitor cells (EPCs) [11], the proliferation and invasion of tumors [12,13], and the injury of embryonic stem cell-derived neurons [14]. However, the function and mechanism of miR-107 in the differentiation and adipogenesis of bovine adipocyte has not been established. In a previous study, we obtained 412 mature miRNAs in adipose tissue of bovine through Sorexa SBS technology sequencing [15]. We focused on miR-107(bta-miR-107) for its high expression. miR-107 was located in intron 7 of *PANK1* (Pantothenate Kinase 1) on chromosome 26 of cattle, and its sequence is highly conserved among different species [10].

Apolipoprotein C-2 (*APOC2*) is an exchangeable apolipoprotein that is found in triglyceride-rich lipoproteins (TRL), such as chylomicrons (CM), high-density lipoprotein (HDL), and very low-density lipoproteins (VLDL). APOC2 acts as a cofactor of lipoprotein lipase and plays a critical role in TRL metabolism [16]. APOC2 is an activator of lipoprotein lipase (LPL), which activates LPL activity, thereby hydrolyzing and removing triglycerides in chylomicrons and VLDL; in the lipoprotein metabolism pathways, high levels of APOC2 hinder VLDL lipolysis and the uptake of VLDL particles by the liver [17]. Studies have shown that *APOC2* is an appropriate nutritional biomarker to measure human health [18] and plays an important role in lipoprotein metabolism. A mutation on exon 3 of the *APOC2* (c.86A > CC) can significantly decrease LPL activity in plasma and significantly increase triglyceride levels in serum, resulting in severe hypertriglyceridemia [19]. In zebrafish, *APOC2* mutants show pancreatic ectopic and overgrowth during embryo development, while yolk depletion and angiogenesis are slightly delayed during larval development [20]. However, the mechanism by which *APOC2* participates in adipocyte proliferation and differentiation is poorly understood.

In this study, we performed the overexpression and knockdown of miR-107 in adipocytes to explore the mechanism of miR-107 on adipocyte differentiation and adipogenesis. These findings will provide a valuable regulatory resource for understanding the mechanism of miRNAs’ function during adipogenesis.

## 2. Materials and Methods

### 2.1. Tissues Collected and RNA Isolation

Bovine subcutaneous fat tissue samples (*n* = 3) and 5 other tissues (muscle, heart, spleen, kidney, and liver) were collected from three adult female Nanyang cattle aged 26 months for RNA extraction at a local slaughterhouse in Xinyang. All tissues were rapidly frozen in liquid nitrogen and stored at −80 °C until RNA was extracted. Furthermore, the primary adipocytes were isolated immediately with phosphate-buffered saline (PBS) containing 1.5% dual antibiotics (streptomycin and penicillin) after the subcutaneous fat tissue of the cattle was collected and brought back to the laboratory. Total RNA was extracted from tissues or cells by TRIzol reagent (TaKaRa, Dalin, China). All animal samples and protocols used in this experiment were approved by the Animal Care and Use Committee of Xinyang Normal University.

### 2.2. Real-Time Quantitative PCR (RT-qPCR)

Total RNA was extracted from tissues or cells, then cDNA was synthesized using the PrimeScript™ RT reagent Kit (TaKaRa, Dalin, China), following the manufacturer’s instructions. The miR-107 stem–loop primer (for reverse-transcribed cDNA) and other qPCR primers were designed using software primer 5.0 (Table 1). RT-qPCR was performed with the SYBR Green PCR Master Mix Regent Kit (TaKaRa). The fold change in the gene expression was detected by the 2^−ΔΔCt^ method and normalized with β-actin and U6.

### 2.3. Vector Construction

In order to overexpress and interfere with miR-107 (AGCAGCAUUGUACAGGGCUAUC) in adipocytes, agomiR-107 and antagomiR-107 were synthesized in RiboBio Co., Ltd., (Guangzhou, China). The APOC2-3′UTR sequence, including the miR107 binding site, was cloned using a pair of primers: (forward) 5′-CCGCTCGAGCAGGGATTATTACTGACCAGG- 3′, (reverse) 5′-ATAAGAATGCGGCCGCGCTAGAATTGGGAACAGGATG-3′. Additionally, the sequence of APOC2 3′UTR with a 4-base deletion in the miR-107 binding site (APOC2-3′UTR-Mut) was cloned by mutagenic primers: 5′-CTGACCAGGTCTTTTCTGTGTCAGGAAAAGATTAA-3′ and 5′-CACAGAAAAGACCTGGTCAGTA-3. Then, the recombinant psi-CHECK-2- *APOC2*-3′UTR/-Mut dual-luciferase reporter vector was constructed using restriction enzymes *Not* I and *Xho* I (TaKaRa, Dalin, China). In addition, the overexpression vector pcDNA3.1-APOC2 was constructed using the restriction enzyme *Hind* III/*Kpn* I (TaKaRa, Dalin, China) (Table 2), and the siRNA for the knockdown, *APOC2* expression was synthesized by RiboBio Co., Ltd., (Guangzhou, China).

### 2.4. Cell Culture and Differentiation

Primary adipocytes were isolated from subcutaneous fat tissues of Nanyang cattle using the tissue block isolation method. The details on cell isolation and culture conditions have been described previously [21]. For adipocyte differentiation assays, cells were cultured using DMEM with 10% FBS in 6-well plates. After reaching approximately 90% confluence, the cells were treated with a differentiation induction medium (1 μM dexamethasone, 0.5 mM IBMX, and 1 μM rosiglitazone, 10 μg/mL insulin). Then, the cells were cultured with a differentiation medium (1 μM rosiglitazone, 10 μg/mL insulin) 48 h later.

### 2.5. Oil Red O Staining

The adipocytes were stained for 20 min using Oil Red O solution (PBS with 60% isopropanol and 0.3% Oil Red O) after fixation in 4% paraformaldehyde for 30 min. Then, the stained cells were eluted with isopropanol and analyzed via a quantitative spectrophotometer at 490 nm OD.

### 2.6. Cell Proliferation Assay

Cell proliferation was examined by the Cell Counting Kit-8 (CCK-8) assay (Multisciences, Hangzhou, China). The cells were cultured in 96-well plates, and six independent replicates were set for each group. After 48 h, 10 μL of CCK-8 reagent per well was added, then incubated at 37 °C for 4 h. The OD value of each sample was detected at 450 nm using a microplate reader (Molecular Devices, Sunnyvale, CA, USA). Furthermore, we assessed cell proliferation through an EdU incorporation assay (Ribobio, Guangzhou, China). The experimental procedures were carried out according to the manufacturer’s instructions on the Cell-Light EdU DNA cell proliferation kit. Three independent replicates were set for each treatment group.

### 2.7. Western Blot Analysis

We collected cystoplasmatic protein from the different groups with RIPA buffer (Solarbio, Beijing, China) containing 1 mM PMSF (Solarbio, Beijing, China) and centrifugation. BCA Kit (Solarbio, Beijing, China) was used to detect protein concentration. The proteins were boiled with SDS loading buffer at 100 °C for 15 min and then loaded and separated with 10% SDS-polyacrylamide gel electrophoresis. Subsequently, the proteins were transferred to 0.2 μm polyvinylidene fluoride (PVDF) membranes and blocked in TBST buffer with 5% skim milk for 2 h, and then sequentially incubated with primary antibodies and secondary antibodies. β-actin was used as the internal control. Finally, antibody-reacting bands were exposed to ECL luminous fluid (Solarbio, Beijing, China).

### 2.8. Statistical Analysis

All data in this study are presented as mean ± SEM (standard error of the mean), and the Student’s t-test was used to determine differences between groups. *p* < 0.05 was considered to be statistically significant. The gray value of the gels image was analyzed by the software Image J.

## 3. Results

### 3.1. miR-107 Expression Profiles in Different Tissues

**Figure 1:**
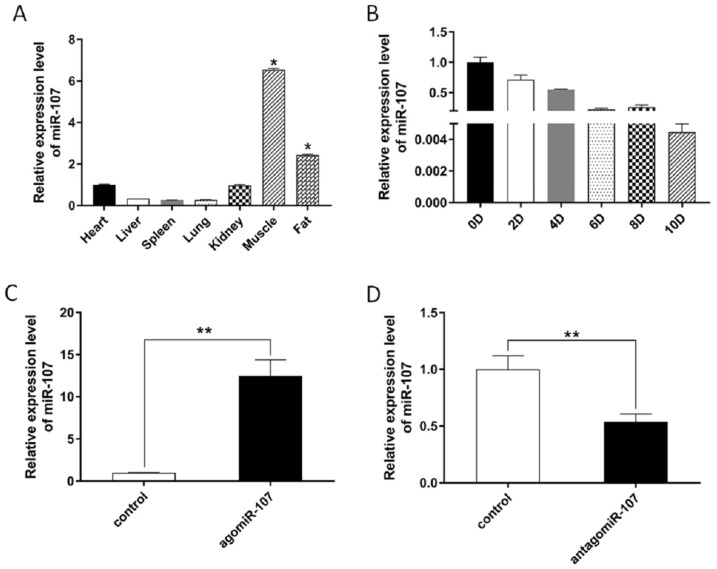
**Expression profiles of miR-107 detected by RT-qPCR.** (**A**) Expression of miR-107 in different tissues of Nanyang cattle. (**B**) Expression of miR-107 at different stages of adipocyte differentiation. 0D–10D represent day 0 to day 10 of cell differentiation, respectively. (**C**,**D**) Transfection efficiency of agomiR-107 and antagomiR-107 were detected by RT-qPCR in bovine adipocytes. Values are mean ± SEM, * *p* < 0.05, ** *p* < 0.01.

In this study, we selected miR-107 as the candidate for its high expression level in adipose tissue of bovine (Figure 1A). In addition, we detected the expression of miR-107 at different stages of adipocyte differentiation, and the results showed that miR-107 expression was decreased during adipocyte differentiation (Figure 1B). These results may indicate that miR-107 functions as a negative regulator during bovine adipogenesis. In order to further investigate the function of miR-107, we synthesized agomiR-107 and antagomiR-107 for miR-107 overexpression and interference. The transfection efficiency of agomiR-107 and antagomiR-107 in bovine primary adipocytes was detected by RT-qPCR, and the results showed a significant difference in miR-107 expression (Figure 1C,D), which provided a basis for further studies.

### 3.2. miR-107 Suppressed Adipocyte Differentiation

**Figure 2:**
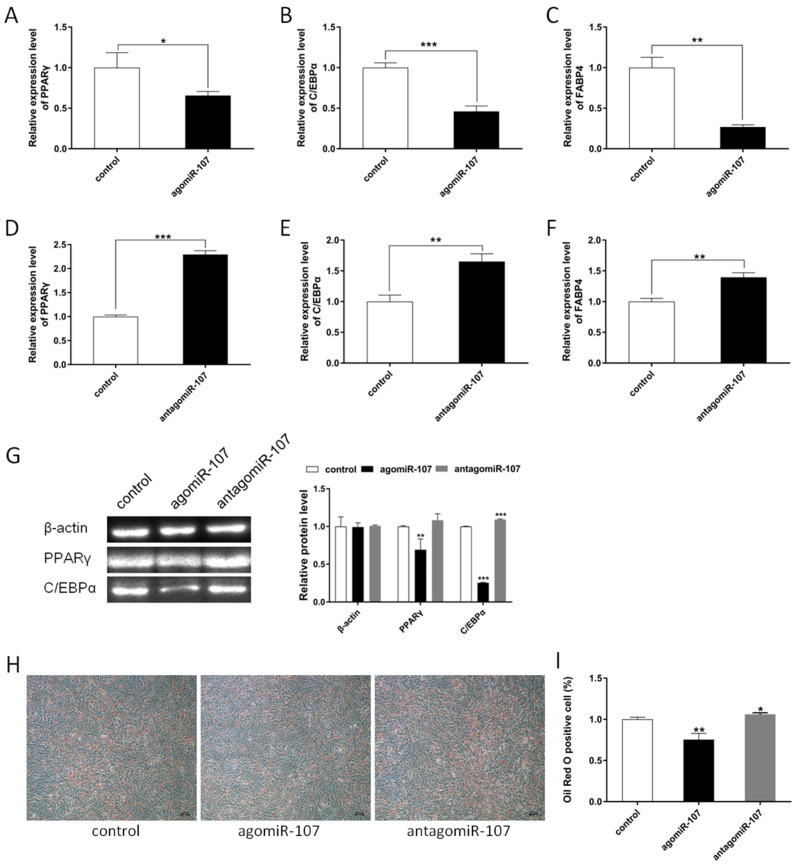
**miR-107 suppressed adipocyte differentiation.** (**A**–**C**) Expression levels of *PPARγ*, *C/EBPα*, and *FABP4* were detected in adipocytes with miR-107 overexpression. (**D**–**F**) Expression levels of *PPARγ*, *C/EBPα*, and *FABP4* were detected in adipocytes after miR-107 interference. (**G**) The protein levels of PPARγ and C/EBPα were detected by a Western blot in adipocytes of miR-107 overexpression and interference. (**H**,**I**) Oil Red O staining was performed to observe the fat deposition, and stained adipocytes were eluted with isopropanol and analyzed via quantitative spectrophotometer at 490 nm OD. Values are mean ± SEM, * *p* < 0.05, ** *p* < 0.01, *** *p* < 0.001.

To determine whether the miR-107 is involved in fat deposition, we used agomiR-107 or antagomiR-107 to transfect the adipocytes. Then, the expression of established adipogenic markers, *PPAR*γ, *C/EBP*α, and FABP4, was detected by RT-qPCR. The results showed that miR-107 suppressed the mRNA level of *PPAR*γ, *C/EBP*α, and FABP4 (Figure 2A–C), while the antagomiR-107 promoted the expression of *PPAR*γ, *C/EBP*α, and *FABP4* (Figure 2D–F). A Western blot was performed to detect the protein expression levels and the results showed that miR-107 suppressed the protein levels of *PPAR*γ and *C/EBP*α in adipocytes (Figure 2G). Meanwhile, Oil Red O staining was performed to observe the fat deposition. The results showed that a significant decrease in fat deposition was observed at agomiR-107 transfected adipocytes, whereas a strong increase in fat deposition was found at antagomiR-107 treated adipocytes (Figure 2H). Stained adipocytes were further eluted with isopropanol and analyzed via quantitative spectrophotometer at 490 nm OD, which indicated that miR-107 was able to cause a ~20% inhibition of adipogenesis (Figure 2I). From all the above results, we deduced that miR-107 suppressed adipocyte differentiation.

### 3.3. miR-107 Suppressed Adipocyte Proliferation

**Figure 3:**
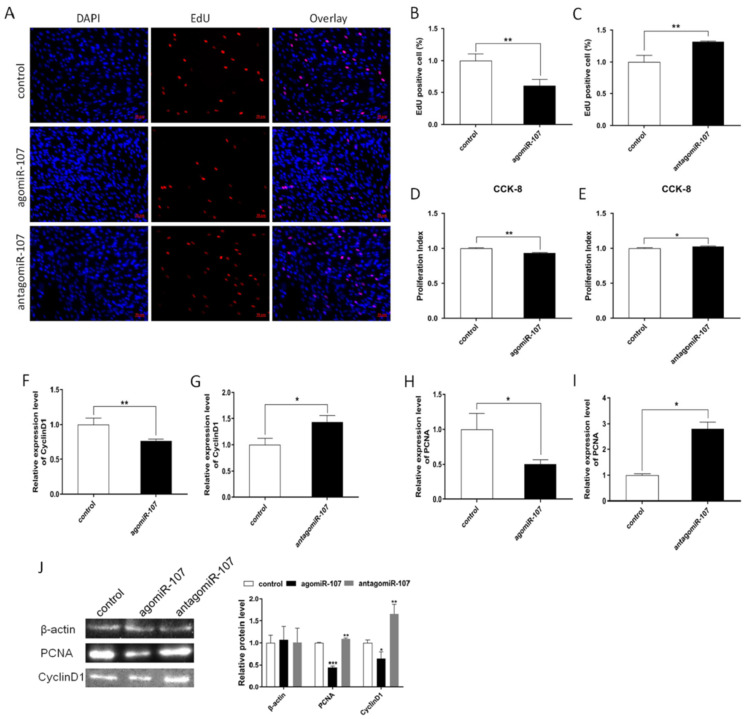
**miR-107 suppressed adipocyte proliferation.** (**A**–**C**) EdU detected adipocyte proliferation index and EdU positive cells analysis. Scale bar: 50 μm. (**D**,**E**) CCK-8 detected adipocyte proliferation index. (**F**–**I**) Expression of the proliferation marker genes *CyclinD1* and *PCNA* were detected by RT-qPCR. (**J**) Protein levels of *CyclinD1* and *PCNA* were detected by a Western blot. Values are mean ± SEM, * *p* < 0.05, ** *p* < 0.01, *** *p* < 0.001.

To determine the role of miR-107 on bovine adipocyte proliferation, adipocytes were transfected with agomiR-107 for overexpression and antagomiR-107 for interference expression. Then, we performed a 5-ethynyl-2′-deoxyuridine (EdU) assay, Cell counting kit-8 (CCK-8) assay, RT-qPCR, and Western blotting assays to detect cell proliferation. The EdU assay showed that overexpression of miR-107 could decrease the number of EdU positive cells (Figure 3A,B), while antagomiR-107 increased the number of EdU positive cells (Figure 3A,C). The CCK-8 assay revealed that miR-107 inhibited cell viability significantly (Figure 3D,E). Moreover, we detected the expression of cell proliferation-related genes, *CyclinD1* and *PCNA*. We found that miR-107 could significantly inhibit the mRNA and protein level expression of these genes (Figure 3F,H,J), while antagomiR-107 could promote *CyclinD1* and *PCNA* expression in both mRNA and protein levels (Figure 3G,I,J). These results suggested that miR-107 suppressed adipocyte proliferation.

### 3.4. miR-107 Directly Targeted APOC2

**Figure 4:**
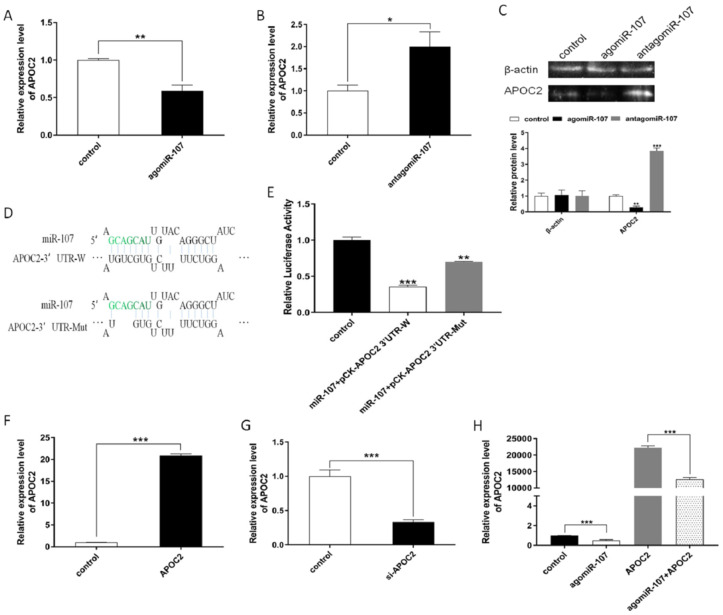
**miR-107 directly targeted *APOC2* 3′UTR.** (**A**,**B**) The mRNA expression level of *APOC2* was detected by RT-qPCR in the miR-107 overexpression and interference groups. (**C**) Protein levels of *APOC2* were detected by a Western blot. (**D**) Sequence of miR-107 and its predicted binding site in *APOC2* 3′UTR and mutation 3′UTR. (**E**) miR-107 co-transfected with pCK-APOC2 3′UTR-W and pCK-APOC2 3′UTR-Mut into bovine adipocytes individually, and Renilla luciferase activity was normalized to the firefly luciferase activity. (**F**,**G**) Transfection efficiency of *APOC2* and si-APOC2 was detected by RT-qPCR in bovine adipocytes. (**H**) *APOC2* expression in adipocytes was detected in groups of individually transfected *miR-107* and *APOC2* and co-transfected *miR-107* and *APOC2* groups. Values are mean ± SEM, * *p* < 0.05, ** *p* < 0.01, *** *p* < 0.001.

In order to explore the regulatory mechanism of miR-107 in adipocyte differentiation, we performed a bioinformatics analysis for selected potential target genes of miR-107 using the TargetScan6.2 and RNAhybrid software. *APOC2* was selected as a potential target gene of miR-107 for its highly conserved miR-107-complementary binding site in the mRNA 3′UTR. RT-qPCR and a Western blot were performed to detect *APOC2* expression, and we found that miR-107 markedly suppressed the expression of *APOC2* at both the mRNA and protein levels (Figure 4A,C), while antagomiR-107 significantly increased the expression of *APOC2* (Figure 4B,C). We performed a dual luciferase activity assay to further elucidate the possible association of miR-107 with APOC2. The result revealed that Renilla luciferase activity was significantly decreased in the group of co-transfected agomiR-107 and pCK-APOC2-3′UTR-W compared to the group of con-transfected agomiR-107 and pCK-APOC2-3′UTR-Mut (Figure 4D,E). Therefore, we concluded that miR-107 was inhibiting *APOC2* expression via directly targeting it. In order to further investigate the function of *APOC2* in adipocytes, we constructed a pcDNA3.1-APOC2 vector for *APOC2* overexpression and synthesized siRNA for *APOC2* interference. The transfection efficiency showed significantly different expressions of *APOC2* (Figure 4F,G). In addition, we performed the salvage experiment and found a significant increase in *APOC2* expression after overexpression of pcDNA-APOC2; co-transfection of miR-107 could significantly reduce *APOC2* expression levels (Figure 4H).

### 3.5. APOC2 Regulated Adipocyte Differentiation and Proliferation

**Figure 5:**
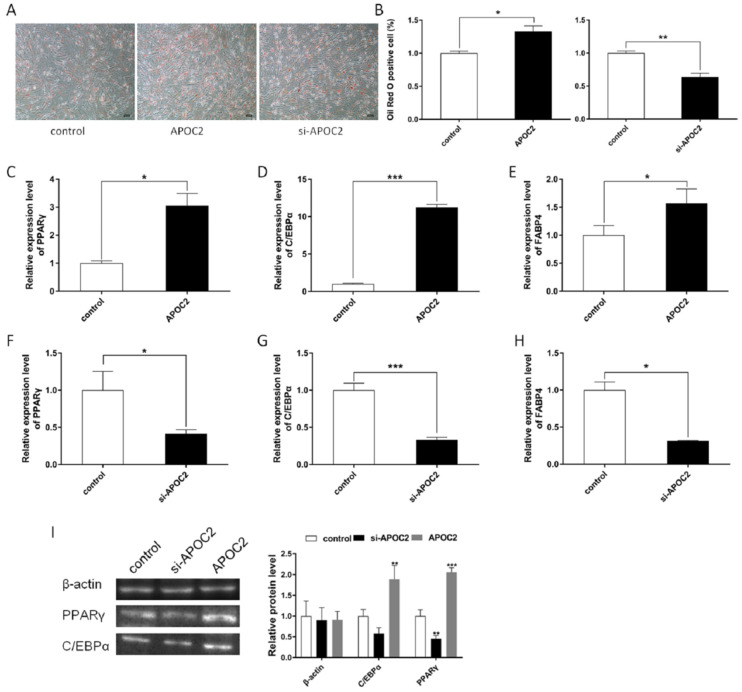
***APOC2* promotes adipocyte differentiation.** (**A**) Oil Red O staining was performed to observe the fat deposition. (**B**) Oil Red O positive cells were evaluated via a quantitative spectrophotometer at 490 nm OD. (**C**–**E**) Expression levels of *PPARγ*, *C/EBPα,* and *FABP4* were detected in adipocytes with *APOC2* overexpression. (**D**–**H**) After siRNA interference with *APOC2*, the expression levels of *PPARγ*, *C/EBPα,* and *FABP4* in adipocytes were detected by RT-qPCR. (**I**) Protein levels of PPARγ and C/EBPα were detected by a Western blot. Values are mean ± SEM, * *p* < 0.05, ** *p* < 0.01, *** *p* < 0.001.

To investigate the role of *APOC2* in adipocyte differentiation, we overexpressed and/or knocked down *APOC2* using pcDNA3.1-APOC2 and siRNA in preadipocytes. An Oil Red O staining assay was performed to observe the fat deposition, and the results showed that *APOC2* increased the formation of lipid droplets in adipocyte differentiation while si-APOC2 inhibited the production of lipid droplets (Figure 5A,B). In addition, we detected the expression of *PPAR*γ, *C/EBP*α, and *FABP4*, which are marker genes for adipogenesis, and found that overexpression of *APOC2* significantly increased mRNA expression of *PPAR*γ, *C/EBP*α, and *FABP4* (Figure 5C–E); conversely, si-APOC2 significantly suppressed their expression (Figure 5F–H), and the results of protein expression detected by Western blotting were consistent with mRNA expression (Figure 5I).

**Figure 6:**
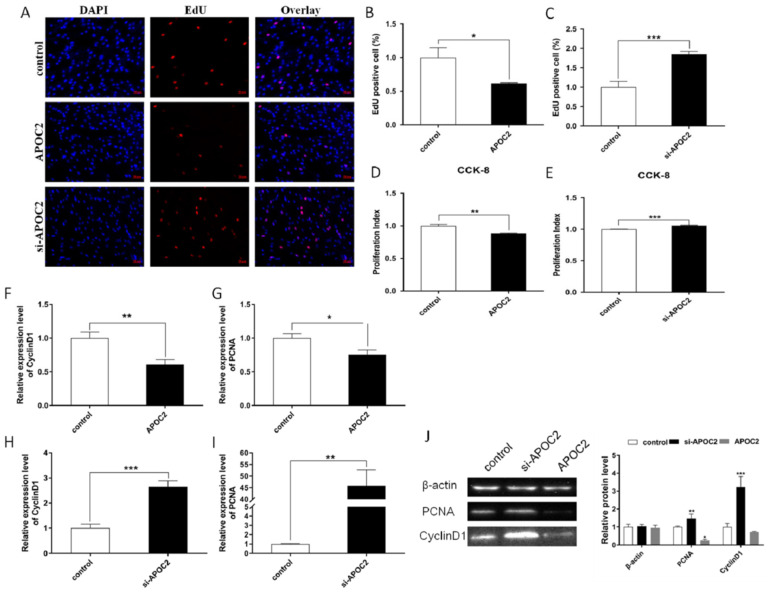
***APOC2* suppresses adipocyte proliferation.** (**A**–**C**) EdU detected adipocyte proliferation and analysis of EdU-positive cells. Scale bar: 50 μm. (**D**,**E**) CCK-8 detected adipocyte proliferation index. (**F**–**I**) Expression of proliferation marker genes *CyclinD1* and *PCNA* were detected by RT-qPCR. (**J**) Protein levels of *CyclinD1* and *PCNA* were detected by a Western blot. Values are mean ± SEM, * *p* < 0.05, ** *p* < 0.01, *** *p* < 0.001.

For cell proliferation, the EdU staining and CCK-8 assay results revealed that *APOC2* strongly suppressed adipocyte proliferation (Figure 6A,B,D), while *si-APOC2* promoted adipocyte proliferation (Figure 6A,C,E). Meanwhile, we detected the expression of *CyclinD1* and *PCNA* in both mRNA and protein levels, and the results of RT-qPCR showed that *APOC2* significantly inhibited *CyclinD1* and *PCNA* mRNA expression (Figure 6F,G). Conversely, *si-APOC2* increased the expression of *CyclinD1* and *PCNA* (Figure 6H,I). Furthermore, we performed a Western blot assay to detect the expression of *CyclinD1* and *PCNA* in protein levels, and the results were consistent with the mRNA expression (Figure 6J). These results demonstrated that *APOC2* regulated adipocyte proliferation and differentiation.

In conclusion, miR-107 was inhibiting *APOC2* expression via directly targeting it, and the effect of si-APOC2 on adipocytes was consistent with the miR-107 overexpression, so we concluded that miR-107 regulates adipogenesis by targeting *APOC2*.

## 4. Discussion

Recent studies have highlighted the importance of non-coding RNAs in diverse biological processes, such as miRNAs, lncRNAs, and circRNAs, most specifically in regulating cell differentiation and proliferation. Current hotspots of miRNAs research mainly focus on physiogenesis, tumorigenesis, and cell differentiation, while the mechanism of miRNAs in adipogenesis is largely unknown. The expression levels of miRNAs are found to be more tissue- and cell-type-specific than those of protein-coding genes, and miRNAs have been shown to be differentially expressed across various stages of differentiation. In this study, miRNAs related to cattle adipose development were analyzed, and it was found that miR-107 expression was high in adipose tissue and suppressed adipocyte differentiation by regulating *APOC2*. miRNAs have been reported to be involved in tumorigenesis [22], myoblast differentiation [6,7,8], kidney development [23], and adipogenesis [4,5,24]. The regulation mechanism of miRNAs is mainly to degrade mRNA or hinder protein translation through binding in target sites. For example, miR-324-5p suppresses the inflammatory response of diabetic vessels by degrading the mRNA of *CPT1A,* which was the target gene of miR-324-5p [25]; miR-552 promotes laryngocarcinoma cell progression by directly targeting p53 mRNA 3′-UTR and degrading the mRNA of p53 [26]; miR-378a-3p promotes myoblasts differentiation by targeting *HDAC4* and hinders protein translation [8].

In this study, we identified that miR-107 was downregulated during adipocyte differentiation, and it was highly conserved among different species. It was previously reported that miR-107 may be of particular importance in metabolic diseases [27], be important in regulating neural injury in cortical neurons [14], suppress esophageal squamous cell carcinoma proliferation, migration, and invasion by targeting *Cdc42* [28], and inhibit myoblasts differentiation via *Wnt3a* [10]. However, the effects of miR-107 on adipocyte differentiation and adipogenesis have not been reported. In this study, we performed Oil Red O staining, RT-qPCR, and Western blotting to provide evidence that miR-107 suppresses adipocyte differentiation. The role of miR-107 in adipocyte differentiation was consistent with myoblasts differentiation [10], suggesting that miR-107 has similar functions in different cell types. Moreover, miR-107 inhibition of adipocyte proliferation was proved through CCK-8, EdU assays, RT-qPCR, and Western blotting, which is consistent with the effect in cancer cells [28].

**Figure 7:**
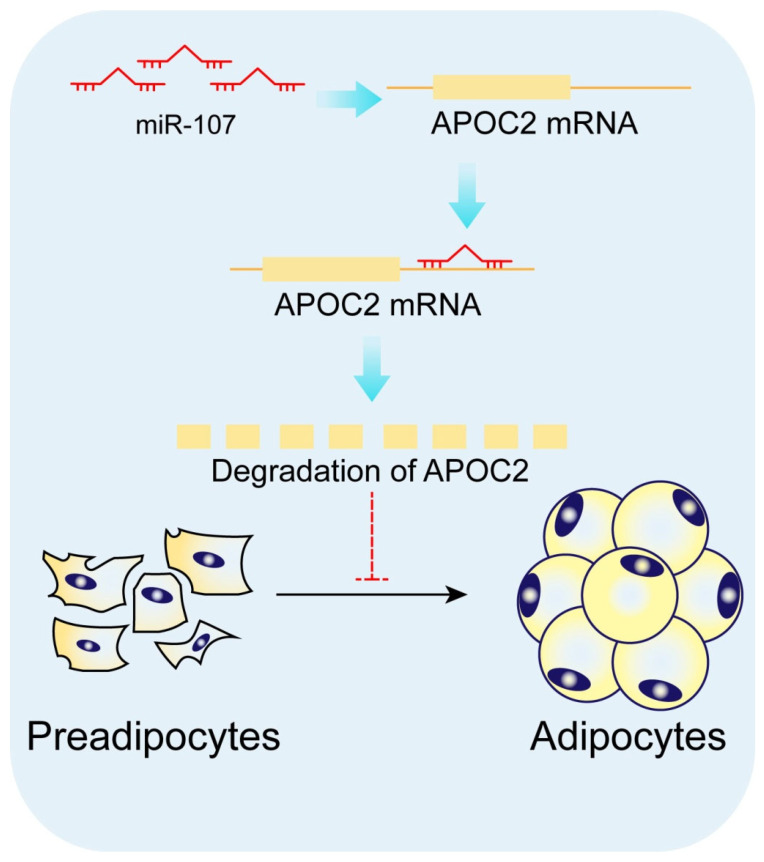
**A model of miR-107 functioning in adipocytes.** miR-107 promotes adipocyte differentiation by regulating APOC2 expression both in mRNA and protein levels.

To explore the regulatory mechanism of miR-107 inhibiting adipocyte differentiation, we performed the bioinformatics analysis and found *APOC2* is a potential target of miR-107 for its highly conserved miR-107-complementary binding sites in the mRNA 3′UTR. In this study, we performed dual luciferase reporter assays, RT-qPCR, and Western blotting to provide evidence that miR-107 suppresses adipocyte differentiation by directly targeting *APOC2* and decreases *APOC2* expression at both the mRNA and protein levels in bovine. Studies have shown that *APOC2* plays an important role in lipoprotein metabolism [16], tissue development [20], and measures human health [18]. *APOC2,* as the cofactor for LPL and nutritional marker, plays a crucial role in triglyceride metabolism and accurate assessment of nutritional status unaffected by inflammation [18,19]. *APOC2* deficiency in humans displays decreased levels of cholesterol ester, chylomicronemia [17], and severe hypertriglyceridemia, which is a considerable risk factor for the development of cardiovascular [20]. To investigate the role of *APOC2* in adipocyte differentiation, we overexpressed and/or knocked down *APOC2* using pcDNA3.1-APOC2 and siRNA in preadipocytes, Oil Red O staining, RT-qPCR, and Western blotting results showed that *APOC2* increased the formation of lipid droplets in adipocyte differentiation which was consistent with miR-107 treatments. Therefore, we concluded that miR-107 inhibits adipocyte differentiation by targeting *APOC2* (Figure 7), and it may provide a new molecular target for the future clarification of the regulation mechanism of adipocyte differentiation and lipogenesis in bovine. Numerous studies have shown that the pathogenesis of a series of lipid diseases is closely related to abnormal differentiation and proliferation of adipocytes, abnormal adipogenesis, and lipid metabolisms, such as obesity, hyperlipidemia, hypertriglyceridemia, and chylomicronemia. Thus, it is possible that miR-107 is a candidate molecular marker to be used for the treatment of lipid diseases, such as obesity, and its role needs to be further explored.

## 5. Conclusions

In conclusion, we identified that miR-107 was downregulated during adipocyte differentiation. We performed a series of assays to characterize and evaluate the function of miR-107, and our findings suggested that miR-107 regulates bovine adipocyte differentiation and lipogenesis by directly targeting *APOC2*. In summary, these results provide a theoretical and experimental basis for future clarification of the regulation mechanism of adipocyte differentiation and lipogenesis in bovine. Moreover, miR-107 may be a potential molecular target to be used for the treatment of lipid-related diseases in the future, such as obesity, and its role needs to be further explored.

## Figures and Tables

**Table 1:** Primers for RT-qPCR.

Name	Forward Primer 5′→3′	Reverse Primer 5′→3′
β-actin	CTGGCATTGTCATGGACTCTG	GCTCGGCTGTGGTGGTAAA
PPARγ	AGCCCAAGTTCGAGTTTGCT	TCTTGTATGTCCTCAATGGGCT
C/EBPα	TGGACAAGAACAGCAACGAG	TTGTCACTGGTCAGCTCCAG
FABP4	AAGTCAAGAGCATCGTAA	CCAGCACCATCTTATCAT
CyclinD1	CGAGCACTTCCTCTCCAAGA	AATGAACTTCACGTCTGTGGCA
PCNA	TCCAGAACAAGAGTATAGC	TACAACAGCATCTCCAAT
AOPC2	ATACAGCAAGAGCACGGCA	TCCTGACAGCACAGAAAAGACC
U6	CTCGCTTCGGCAGCACA	AACGCTTCACGAATTTGCGT
miR-107	ggAGCAGCATTGTACAGGGCTATC	GCAGGGTCCGAGGTATTC
miR-107 stem-loop primer	gtcgtatccagtgcagggtccgaggtattcgcactggatacgacGATAGC	

**Table 2:** Primers for the vector construction.

Name	Forward Primer 5′→3′	Reverse Primer 5′→3′
APOC2-CDS	CCAAGCTTGGAGAGTCTGCCACCTCAGTGTT	CGGAATTCCGGGCTAGAATTGGGAACAGGA
APOC2-3′UTR	CCGCTCGAGCAGGGATTATTACTGACCAGG	ATAAGAATGCGGCCGCGCTAGAATTGGGAACAGGATG
APOC2-3′UTR-Mut-1	GCTGAGGACAGCCGCC	CACAGAAAAGACCTGGTCAGTA
APOC2-3′UTR-Mut-2	CTGACCAGGTCTTTTCTGTGTCAGGAAAAGATTAA	GATAAGTCAGGCTAGAATTGGGAAC
